# Percutaneous treatment of patients with heart diseases: selection, guidance and follow-up. A review

**DOI:** 10.1186/1476-7120-10-16

**Published:** 2012-03-27

**Authors:** Carla Contaldi, Maria-Angela Losi, Antonio Rapacciuolo, Maria Prastaro, Raffaella Lombardi, Valentina Parisi, Lucia S Parrella, Carlo Di Nardo, Alessandra Giamundo, Roberto Puglia, Giovanni Esposito, Federico Piscione, Sandro Betocchi

**Affiliations:** 1 Department of Clinical Medicine, Cardiovascular and Immunological Sciences, University Federico II, Naples, Italy

## Abstract

Aortic stenosis and mitral regurgitation, patent foramen ovale, interatrial septal defect, atrial fibrillation and perivalvular leak, are now amenable to percutaneous treatment. These percutaneous procedures require the use of Transthoracic (TTE), Transesophageal (TEE) and/or Intracardiac echocardiography (ICE). This paper provides an overview of the different percutaneous interventions, trying to provide a systematic and comprehensive approach for selection, guidance and follow-up of patients undergoing these procedures, illustrating the key role of 2D echocardiography.

## Introduction

Advances in cardiovascular interventional techniques have allowed percutaneous treatment of conditions that either previously required open operations or have not been amenable to any treatment.

Aortic stenosis (AS) is the most common valvular abnormality in the western world and it is more frequent in elderly patients with comorbidities. Transcatheter Aortic Valve Implantation (TAVI) offers an alternative to patients with severe symptomatic AS and contraindications for surgery or high risk for surgery [1].

Mitral regurgitation (MR) has a prevalence of 1-2% in the general population and there are several percutaneous techniques for the treatment of it, such as direct annuloplasty, indirect annuloplasty-coronary sinus and ventricular remodeling, however only repair by using Mitraclip has been extensively evaluated [2]. Patent foramen ovale (PFO) and atrial septal defects (ASD) are interruption of atrial septum [3] and their percutaneous closure is a safe and accepted alternative to surgery [4-7].

Atrial fibrillation (AF) is the most common cardiac arrhythmias. At present, percutaneous left atrial appendage (LAA) occlusion may be an acceptable option in selected high-risk patients with AF who are not candidates to oral anticoagulation [8].

Para-valvular leaks (PVLs) represent a complication of cardiac valve replacement and their surgical repair is associated with a high mortality and morbidity rate, thus, in selected cases, percutaneous repair can be performed [9]. This review emphasizes particularly the role of 2D echocardiography in selection, guidance and follow up of patients candidates to percutaneous treatment.

## Valvular diseases

### Transcatheter Aortic Valve Implantation TAVI

#### Diagnosis of AS

**Figure 1:**
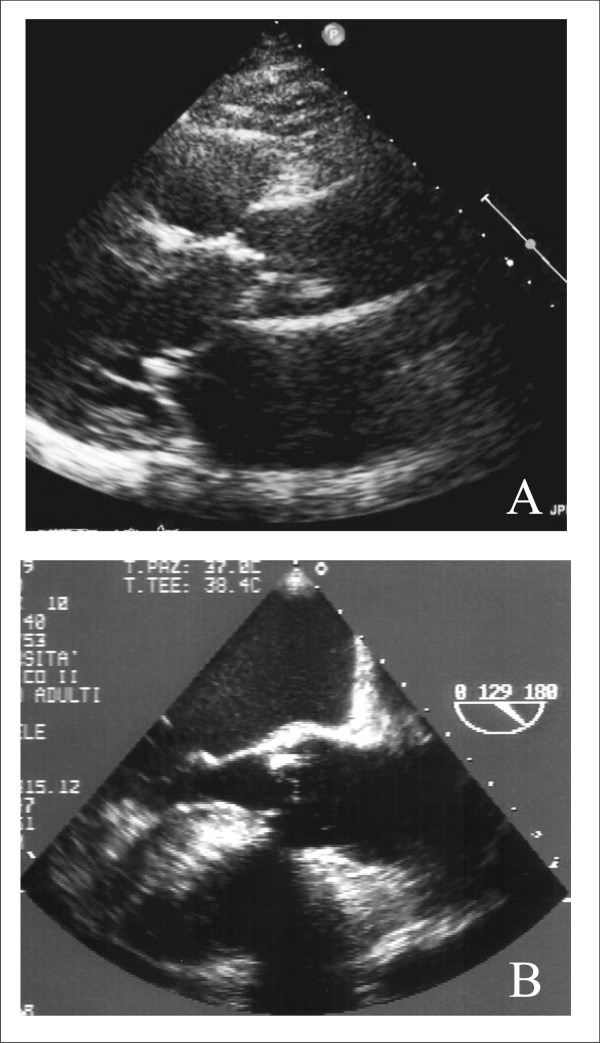
**TTE parasternal long axis (Panel A) and TEE midesophageal long-axis (120°-140°) view (Panel B) useful to measure aortic valve annulus and aorto-tubular junction of a patient with severe AS**.

The severity of AS is usually assessed by TTE according to AHA/ACC [10] and to ESC Guidelines [11] (Figure 1, Panel A and B). Low-dose dobutamine echocardiography can be useful to differentiate between severe and the rare "pseudo severe" AS in patients with low LV ejection fraction and low gradient [10,11].

#### Indications to TAVI

To date, there is a lack of pharmacological therapies to prevent the progression of AS and unfortunately, balloon aortic valvuloplasty has revealed limited long-term efficacy [12].

TAVI can be considered an alternative to surgery for patients with symptomatic severe AS and with contraindications or at high risk for surgery (Table 1) [1]. Patients are defined at high risk for aortic valve surgery if logistic European System for Cardiac Operative Risk Evaluation (EuroSCORE) [13] is > 20% and Society of Thoracic Surgeons (STS) score [14] is > 10%. Currently, there are two prosthetic valves for TAVI: the SAPIEN (Edwards Life Sciences, Inc.) and the CoreValve (Medtronic, Inc.). Edwards-Sapien valve consists of three bovine pericardial leaflets, mounted within a tubular, slotted, stainless steel, balloon-expandable stent [1,15] and it can be introduced by transfemoral or transapical approach. The CoreValve Revalving System has three porcine pericardial leaflets, mounted in a self-expanding, nitinol frame and it can be introduced by transfemoral or trans-subclavian (off-label use) approach [1,15]. Recently, SAPIEN has shown to be superior to medical therapy (including balloon aortic valvuloplasty) [16,17] and to be not inferior to conventional surgery [18]. Pre-procedural screening with TTE assesses heart anatomy and function, and aortic root anatomy. In the first instance it must be verified that the obstruction to left ventricular outflow tract (LVOT) is at valvular level and that AS is severe. The distance from the basal attachment of the non coronary cusp to the basal attachment of the right coronary cusp is measured as aortic valve annulus [15,16], in systole (Table 1). Determining accurate prosthesis size is critical for device stability to minimize paravalvular aortic regurgitation (AR) and to prevent aortic rupture [19]. A gold standard method of annular measurement has yet to be established [1]. TTE is the primary modality [20] for annulus measurement, whereas TEE is performed only if an accurate measurement cannot be made by TTE [16], or if borderline values lead to doubt the feasibility of the procedure [1]. This anteroposterior measurement more closely approximates the minor rather than the major dimension of the elliptically shaped annulus, as measured by multidetector computed tomography (CT) [16]. As current clinical experience and recommendations are based on echocardiographic annular measurements, whereas measurements on CT are generally not routinely used. Height of coronary ostia from the base of the aortic valve leaflets need to be ≥ 10 mm to prevent coronal occlusion when the prosthesis is implanted. In presence of a mitral prosthesis, the prothesis must not be positioned too low, because it can affect mitral prosthetic function [21]. Clinical and echocardiographic indications and contraindications to TAVI are reported in Table 1.

**Table 1:** Indications to TAVI

CLINICAL INDICATIONS	CLINICAL CONTRAINDICATIONS	ECHOCARDIOGRAPHIC INDICATIONS	ECHOCARDIOGRAPHIC CONTRAINDICATIONS
Severe AS with Symptoms and High Risk for Surgery	Life Expectancy < 1 Year Severe Respiratory Insufficiency for Transapical Approach Previous Surgery of the Left Ventricle using a Patch for Transapical Approach	Severe AS 18 < Aortic Annulus < 25 mm for SAPIEN Valve; 20 < Aortic Annulus < 27 mm for CORE Valve Aortic Tubular Junction < 45 mm for CORE Valve	Bicuspid Aortic Valve Subaortic Stenosis Height of coronary ostia from the base of aortic valve leaflets < 10 mm Asymmetric Heavy Aortic Valvular Calcification Intracardiac Thrombus Mitral Regurgitation > 2+ Left Ventricular Ejection Fraction < 20% Severe Left Ventricular Hypertrophy (< 1,7 cm) Bulky Atherosclerosis of the Ascending Aorta and Arch for Transfemoral Approach Calcified Pericardium for Transapical Approach

#### Echocardiography guidance of TAVI

Echocardiography is not mandatory to guide TAVI. Intraprocedural TEE may play a role with the SAPIEN series in guiding valve implantation and in early post implantation assessment [19]. During the transapical approach, TEE is useful to evaluate MR, can increases because of worsening LV function, new wall motion abnormalities or dissyncrony induced by right ventricular pacing. The CoreValve is generally deployed under fluoroscopic guidance, with TEE being used on a discretionary basis, whereas post procedural AR is evaluated by aortography and TTE [1,16]. Device malpositioning can cause severe paraprosthesic leak that can be managed successfully, in selected cases, with implantation of a second device inside the primary prosthesis (Valve-in-Valve procedure) [22].

#### Echocardiographic evaluation of TAVI complications and follow-up

**Figure 2:**
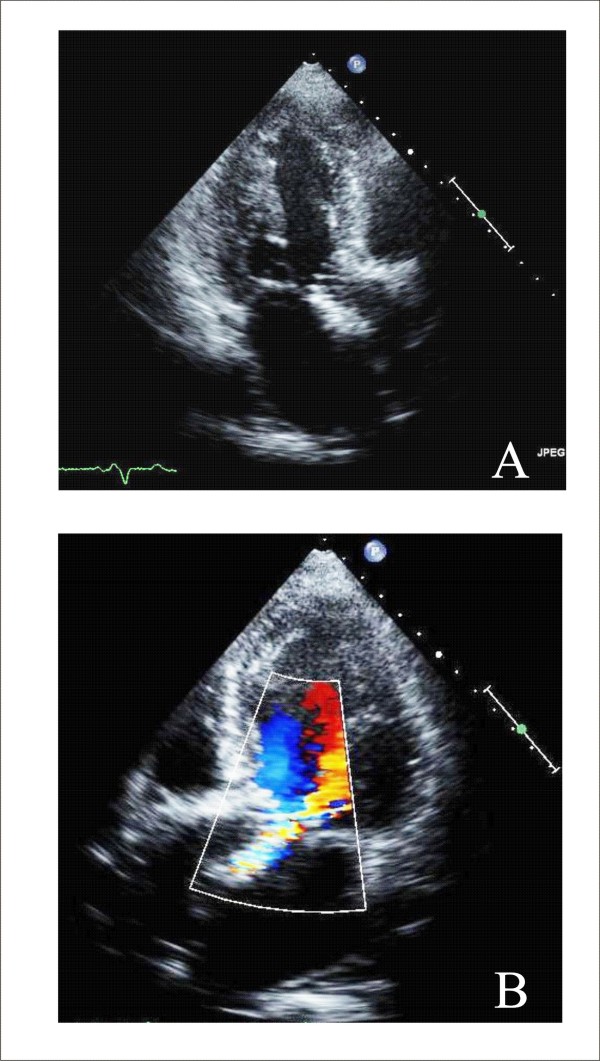
**TTE evaluation of a CoreValve (Panel A) and of a residual mild AR (Panel B)**.

After TAVI, survival at 30 days is 92.9%, at 1 year it is 78.6% and at 2 years it is 73.7%, so midterm to long-term survival is encouraging in high-risk patients, although a substantial proportion of patients died within the first year [23]. During follow-up, TTE is used to evaluate aortic valve area, mean gradient and severity and location of AR (Figure 2, Panel A and B) [16]. During transapical approach, MR can be induced by a partial deformation of the mitral annulus. Trans-prosthetic gradients decrease immediately after successful implantation and remain unchanged over 12 month follow-up [19]. Non severe paravalvular AR, that is seen in the immediate postoperative period, shows improvement in short-term and mid-term follow-up, with only a minority of patients having moderate AR at follow-up at 6 and 12 months [19]. Procedural complications and logistic EuroSCORE are strongly associated with early mortality at 30 days, whereas comorbidities, such as cerebrovascular disease, chronic kidney disease and heart failure, and postprocedural paravalvular AR ≥ 2, mainly impact late outcomes between 30 days and 1 year [24]. Valve-in-Valve patients procedural, 30-day and 12-month outcomes are not different from outcomes of those who underwent the uneventful procedure [22].

### Percutaneous MR repair with the MitraClip system

#### Diagnosis of MR

**Figure 3:**
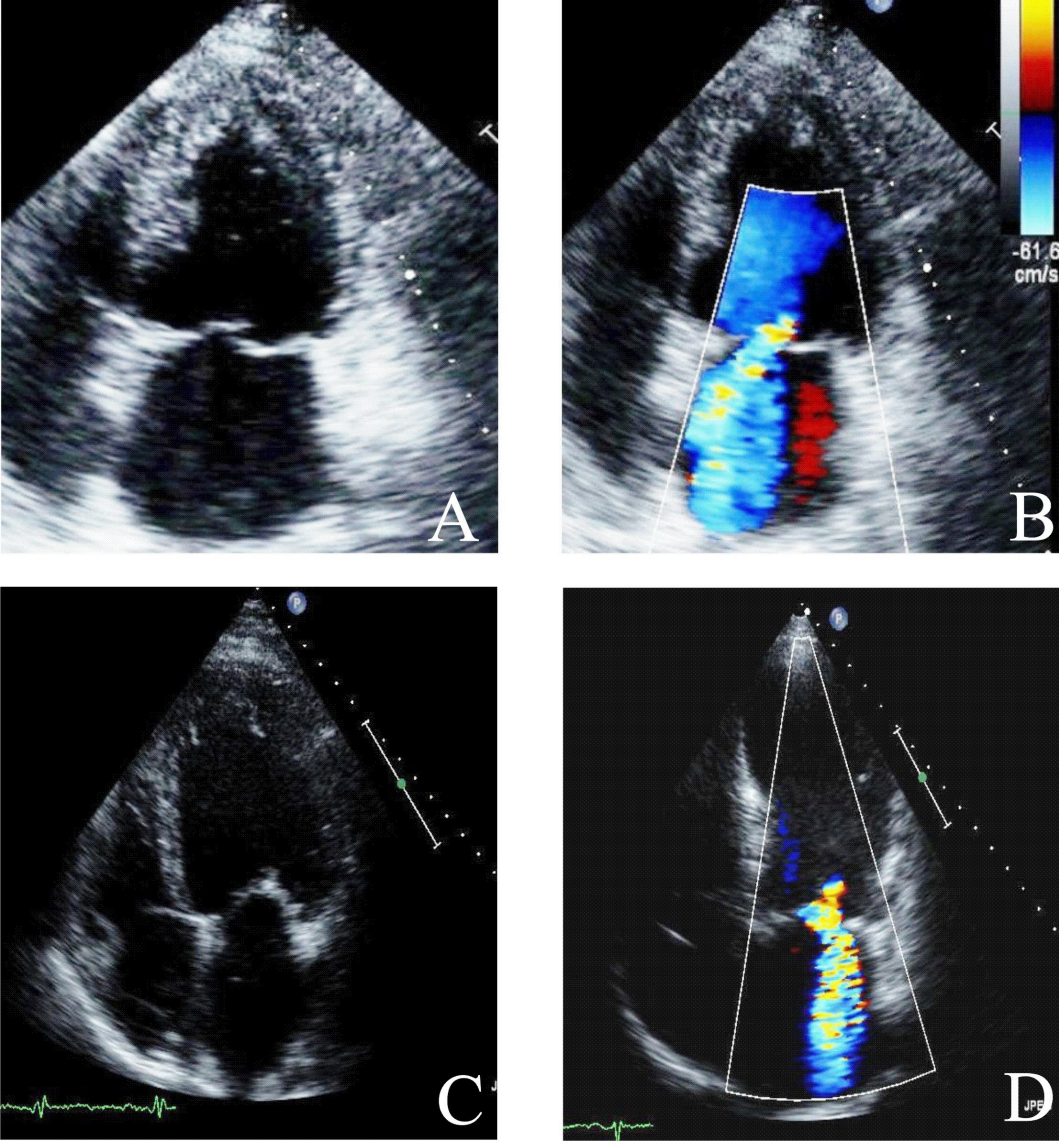
**TTE apical four chamber views without and with color Doppler show severe Organic MR (Panel A and B) and Functional MR (Panel C and D)**.

MR can be divided into primary, or organic, and secondary, or functional categories [25]. Organic MR consists of myxomatus degenerative disease, with leaflets thickened and the cords inappropriately long (Figure 3), whereas in functional MR geometric and/or functional LV change cordal orientation or apical papillary muscles displacing inducing a tethering effect on one or on both leaflets [2] (Figure 3). The grade of MR is done according to the guidelines of the American Society of Echocardiography [26].

#### Indications to MitraClip system

**Figure 4:**
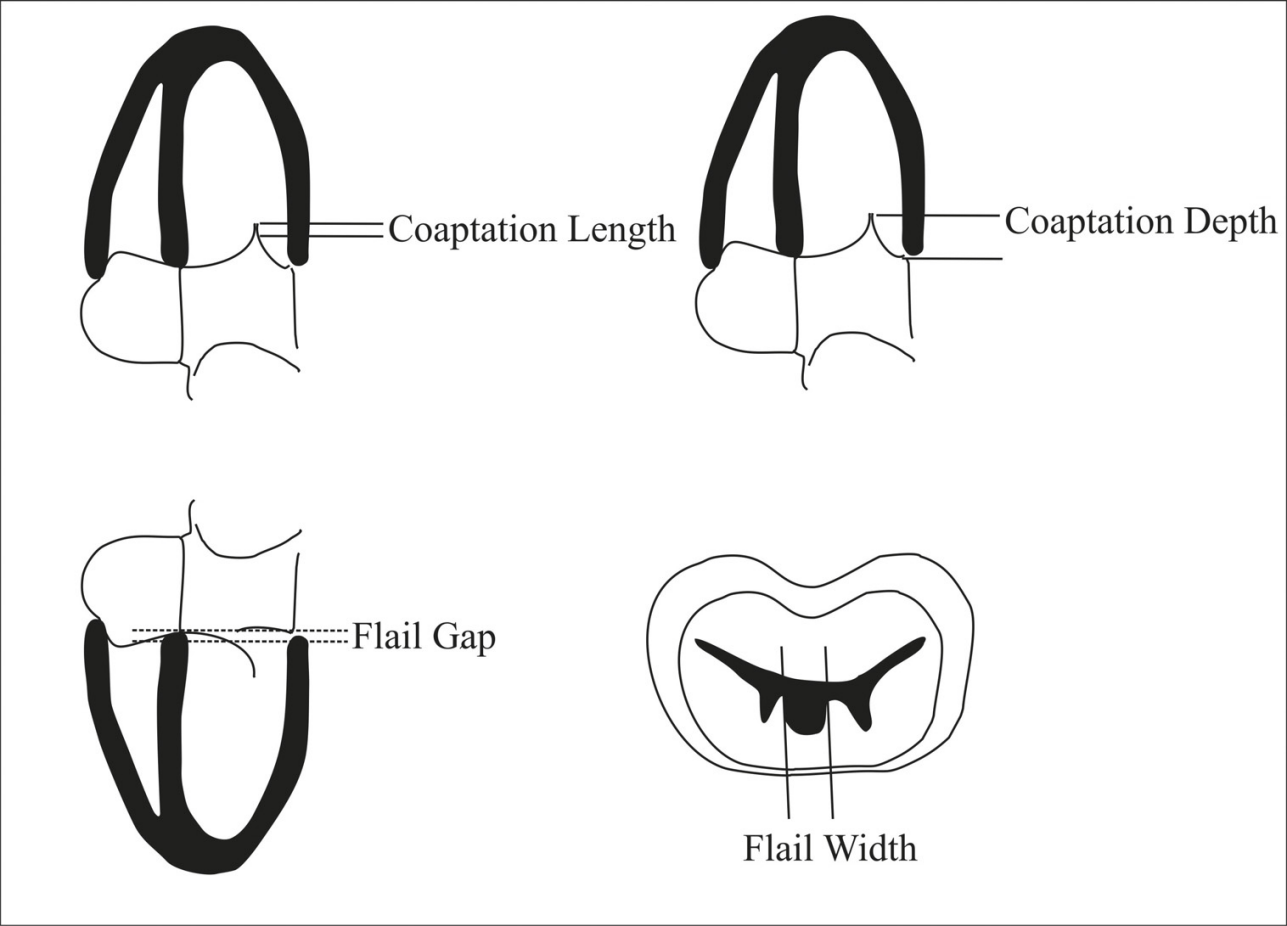
**Scheme of echocardiography measurements to assess for indication to MitraClip System**. Modified by Images courtesy of Abbott Vascular. (c) 2011.

When regurgitation across the valve is the consequence of inappropriate leaflets coaptation, one surgical approach is to create a double orifice by suturing the free edges of the middle leaflet segments, the edge-to-edge, or Alfieri stitch [27]. This technique has now been adapted for transcatheter use which received Conformité Européenne mark approval as the MitraClip (Evalve, Inc., Menlo Park, California) [28]. Indications to intervention with MitraClip system are derived by the Endovascular Valve Edge-to-Edge Repair Study (EVEREST) [29] (Table 2): patients were selected if they met class I indications for intervention according to American College of Cardiology (ACC)/American Heart Association (AHA) recommendations [10]. To assess indications to percutaneous correction, measurements are done by TTE, and TEE (Figure 4) (Table 2). Clinical and echocardiographic indications and contraindications to percutaneous MR repair with MitraClip System are reported in Table 2.

**Table 2:** Indications to MR Treatment by MitraClip System

CLINICAL INDICATIONS	CLINICAL CONTRAINDICATIONS	ECHOCARDIOGRAPHIC INDICATIONS	ECHOCARDIOGRAPHIC CONTRAINDICATIONS
MR 3+ or 4+ and Symptoms or/and New Atrial Fibrillation	Recent Myocardial Infarction Recent Surgical Procedure Renal Insufficiency Endocarditis Rheumatic Heart Disease	MR 3+ or 4+ Compromised Left Ventricular Function (EF < 60% or End-Systolic Diameter ≥ 40 mm) Pulmonary Hypertension A Regurgitant Jet Origin at Level of Coaptation Zone Coaptation Length ≥ 2 mm Coaptation Depth ≤ 11 mm Flail Gap < 10 mm Flail Width < 15 mm	Left Ventricular EF < 25% Left Ventricular End-Systolic Diameter > 55 mm Mitral Valve Orifice Area < 4 cm^2^

#### Echocardiography guidance of percutaneous MR with the MitraClip system

**Figure 5:**
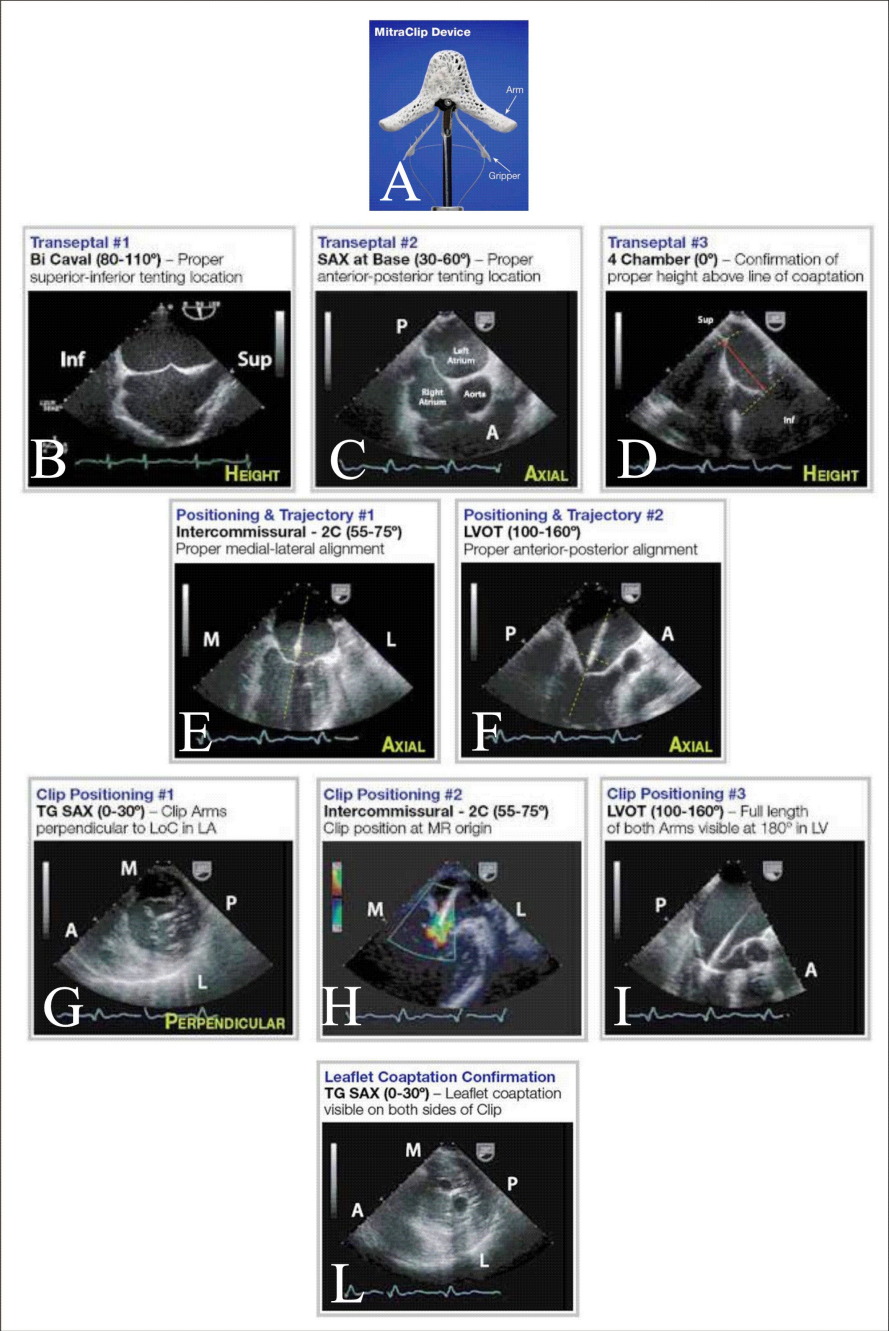
**MitraClip and Echocardiography Guidance of Positioning of MitraClip System**. The clip is a wide cobalt/chromium implant with 2 arms (Panel A). TEE views used to guide transeptal puncture are bicaval view (Panel B), for inferior-superior orientation, basal short-axis view (30°-60°) (Panel C) for anterior-posterior orientation and midesophageal 4-chamber view (Panel D), for assessing height above the valve plane. The clip is moved to center the origin of the MR jet and axially aligned, by the midesophageal intercommissural view or "2 chambers" (60°) (Panel E), and the midesophageal long-axis LV outflow tract view (120-150°) (Panel F). Transgastric short-axis view at the mitral leaflet and subvalvular level (Panel G) is used to rotate the clip to achieve a perpendicular position with the line of coaptation at the middle scallops of the valve at the origin of the MR. Successively, the clip arms are placed, at about 120°, and the mitral leaflets are captured on the arms of the clip. The Color Doppler midesophageal intercommissural view is used to position the clip perpendiculary to MR origin (Panel H). Midesophageal long-axis LV outflow tract view is used to guide grasping and to evaluate leaflet capture (Panel I). Transgastric short-axis view demonstrates a double-orifice mitral valve (Panel L). Images courtesy of Abbott Vascular (c).

The Clip Delivery System (CDS) has the MitraClip, an implantable clip, attached to its distal end (Figure 5) [29,30]. The procedure is performed, with the patient under general anesthesia, by using fluoroscopy and TEE and, on occasion, TTE guidance [29]. The first step is transeptal puncture which requires a crossing of the fossa ovalis in a posterior trajectory toward the line of mitral leaflet coaptation, providing adequate superior clearance above the mitral annular plane. TEE views used for guided transeptal puncture and positioning are reported in Figure 5. After a perpendicular position with the line of coaptation at the middle scallops of the MR origin has been achieved, the clip is advanced and the clip arms are placed in the grasping position and pulled back during systole until the mitral leaflets are captured on the arms of the clip. Intercommissural view with color Doppler is used to position the clip perpendicularly to MR origin (Figure 5), whereas long-axis LV outflow tract view is used to guide grasping and to evaluate leaflet capture, ensuring both leaflets are fully inserted into the clip (Figure 5, Panel I). If leaflet insertion is inadequate, leaflets are released, and clip repositioned. Before deployment, 4- chamber view and commissural view with color Doppler are used for assessment of residual MR and continuous wave Doppler will exclude mitral stenosis. Color Doppler commissural and transgastric short-axis views show clip and two mitral orifices and double-orifice mitral valve with the classic "bow-tie" appearance, respectively (Figure 5). If insufficient improvement in MR is seen after the deployment of one clip, a second MitraClip can be placed [30,31], by assessment of the location of the residual MR [32]. After adequate reduction of MR has been achieved, the clip is deployed, and the CDS and guide catheter are withdrawn [29].

#### Echocardiographic evaluation of percutaneous MR with the MitraClip system Complications and follow-up

In the EVEREST I study, MitraClip had a low incidence of morbidity and mortality and reduction in MR (less than 2+) was observed in the majority of patients [29]. There were no cases of clip embolization, whereas partial clip detachment was seen in 9% of patients; 11% of patients, with acute procedural success, underwent mitral valve surgery; within 1 year, 66% of patients had MR ≤ 2+, demonstrating durability of the percutaneous repair [31].

After percutaneous mitral valve repair, mitral valve area decreases without evidence of clinically significant mitral stenosis [32].

The EVEREST II is the first randomized trial which compares MitraClip device with open mitral valve surgery. Although percutaneous repair was less effective in to reducing MR than conventional surgery, it was associated with superior safety and similar improvements in clinical outcomes at 12 months [32].

TTE follow-up is recommended at 1 and 12 months [33].

## Percutaneous closure of PFO and ASD

### Diagnosis of PFO

**Figure 6:**
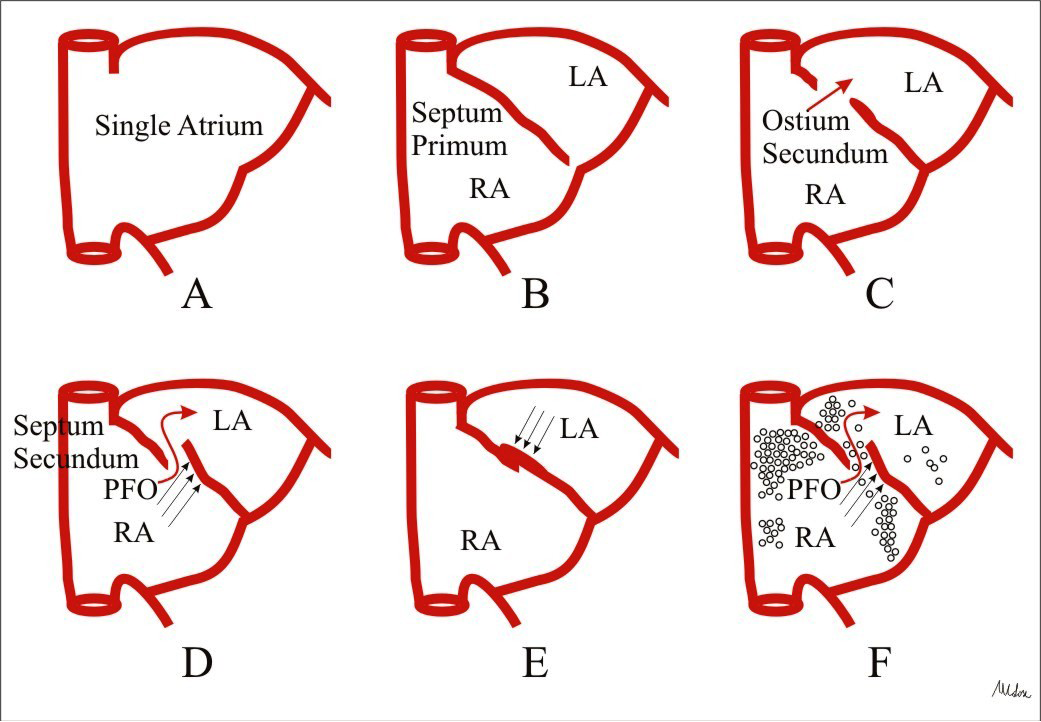
**Interatrial septal development**. The primitive atrium is a single cavity (Panel A) subsequently divided by the septum primum which grows down from the roof of the atrium, toward the developing endocardial cushions (Panel B). Thus, small perforations begin to develop superiorly resulting in the ostium secundum (Panel C). The atrial roof grows down along the right side of the septum primum, the septum secundum, which comes to lie over the ostium secundum, however an opening remains between septa, the PFO (Panel D). At birth, lung pressures drop and the blood pressure in the left atrium exceeds that of the right atrium, so that the septum primum is shoved against the septum secundum, obtaining septa fusion (Panel E). If this final step does not occur, PFO results (Panel F).

**Figure 7:**
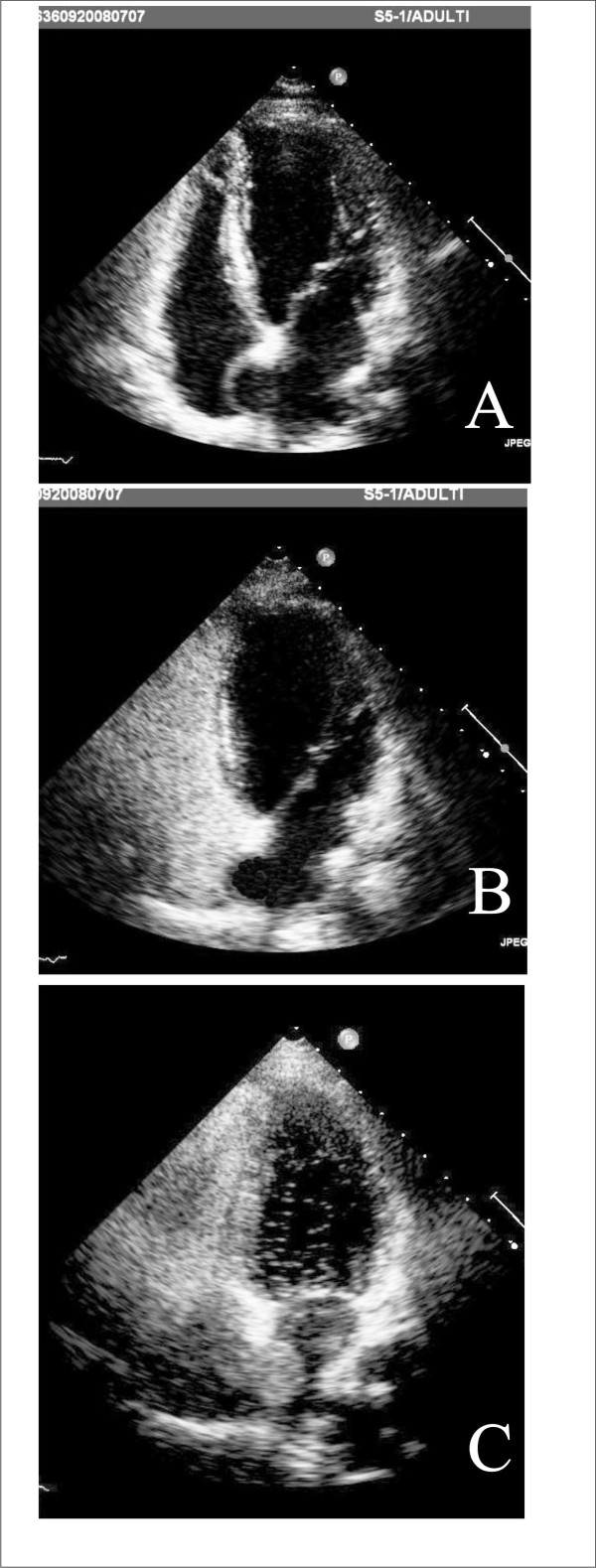
**Panel A: interatrial septum bowing towards the right atrium during normal breathing; Panel B: bowing of the atrial septum towards the left atrium demonstrating that an adequate Valsalva manoeuvre has been performed; Panel C: microbubbles in the left cavities after Valsalva manoeuvre within three cardiac cycles after the contrast material fills the right atrium, positive for PFO**.

**Figure 8:**
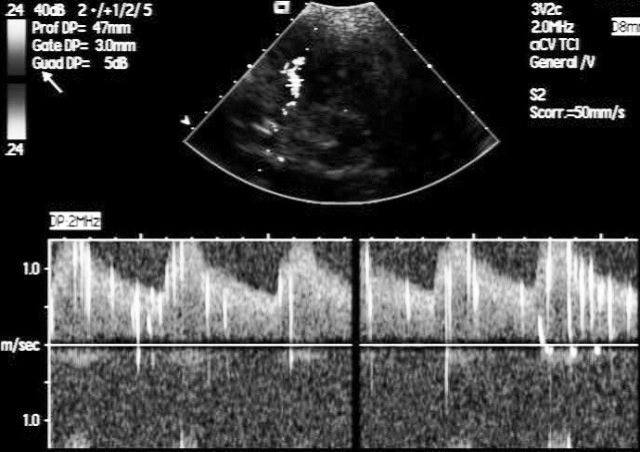
**TCD positive for right-to-left shunt after Valsalva manoeuvre: microbubles overlapping spectrum of Doppler flow velocity of the middle cerebral artery**.

The final step of development of interatrial septum (Figure 6) during fetal life is PFO, functioning as an one way valve; at birth, PFO closes, however if this step does not occur, PFO results, and it will open whenever the pressure in the right exceeds that in the left atrium. Therefore, TTE and TEE, and transcranial Doppler (TCD) visualize PFO opening or, performed with contrast, its functional consequence, the right-to-left shunt (Figure 6, Panel F) [34-36]. Contrast apical 4-chamber view is performed at rest and during Valsalva maneuver, which is considered to be adequate in case a bowing of atrial septum towards left atrium (LA) is visualized (Figure 7) [35]. TTE is considered positive when, at rest or during Valsalva maneuver, after the contrast fills the right atrium, ≥ 3 microbubbles are seen in left chambers within three cardiac cycles [37]; a later appearance of contrast is usually due to intrapulmonary shunts [5]. The numbers of bubbles seen in a single still frame can be used to shunt grading as mild: 3-9 bubbles; moderate: 10-30 bubbles; severe: ≥ 30 bubbles [37,38] (Figure 7). Contrast TCD is an alternative to contrast TTE as a screening test, and when positive, i.e. 3 or more bubbles detected within 20 seconds after the start of injection [39] (Figure 8), a TTE is performed for a comprehensive evaluation of atrial septum such as atrial septal aneurysm (ASA). ASA is diagnosed by a protrusion of septum into left or right atrium of > 10 mm or by the sum of excursions into both atria of > 10 mm with base of ≥ 15 mm [40] (Figure 7, Panel A). Although TEE is considered the gold standard [41], it is usually performed only when a better PFO anatomic definition is needed [42].

### Indications to percutaneous PFO closure

The prevalence of a PFO has been reported to be 24% in the general population and increases to 38% in patients with cryptogenic stroke, suggesting an association between PFO and stroke [43]. Two prospective studies in the general population [43,44] indicate that in healthy people with PFO, embolic events are not more frequent than in controls, therefore primary prevention and echocardiographic screening in asymptomatic patients are not needed [43]. The AHA/ASA and ESO guidelines recommend antiplatelet agents for secondary prevention, while patients with hypercoagulable states or vein thrombosis should be anticoagulated [45-47], whereas when strokes recur, PFO closure is recommended; this comes true also for other high-risk patients, but guidelines leave the definition of "high risk" open [43].

Clinical and echocardiographic indications and controindications to PFO percutaneous closure are reported in Table 3[47-50].

**Table 3:** Indications to PFO Closure

CLINICAL INDICATIONS	CLINICAL CONTRAINDICATIONS	ECHOCARDIOGRAPHIC INDICATIONS	ECHOCARDIOGRAPHIC CONTRAINDICATIONS
Cryptogenic Strokes Migraine with Aura + Thrombophylia and/or Peripheral Venous Thrombosis and/or Previous History of Paradoxical Embolism	Severe Pulmonary Hypertension Recent Gastrointestinal Bleeding Controndications to Antiplatelet or Anticoagulant Therapy Infection at the Time of Implantation	Atrial Septal Aneurysm and/or Large Size of PFO (≥ 4 mm) and/or Long Tunnel of PFO (≥ 1 cm) and/or Severe Right to Left Shunting (> 30 Bubles) and/or Shunting at Rest	Other Congenital Heart Defects Eustachian valve and ChiariNetwork

### Diagnosis of ASD

ASD is a common form of congenital heart disease accounting for approximately 10% of all congenital heart defects [3]. There are four types of ASDs and the only ASD, at moment, susceptible to percutaneous closure is the secundum type [5].

**Figure 9:**
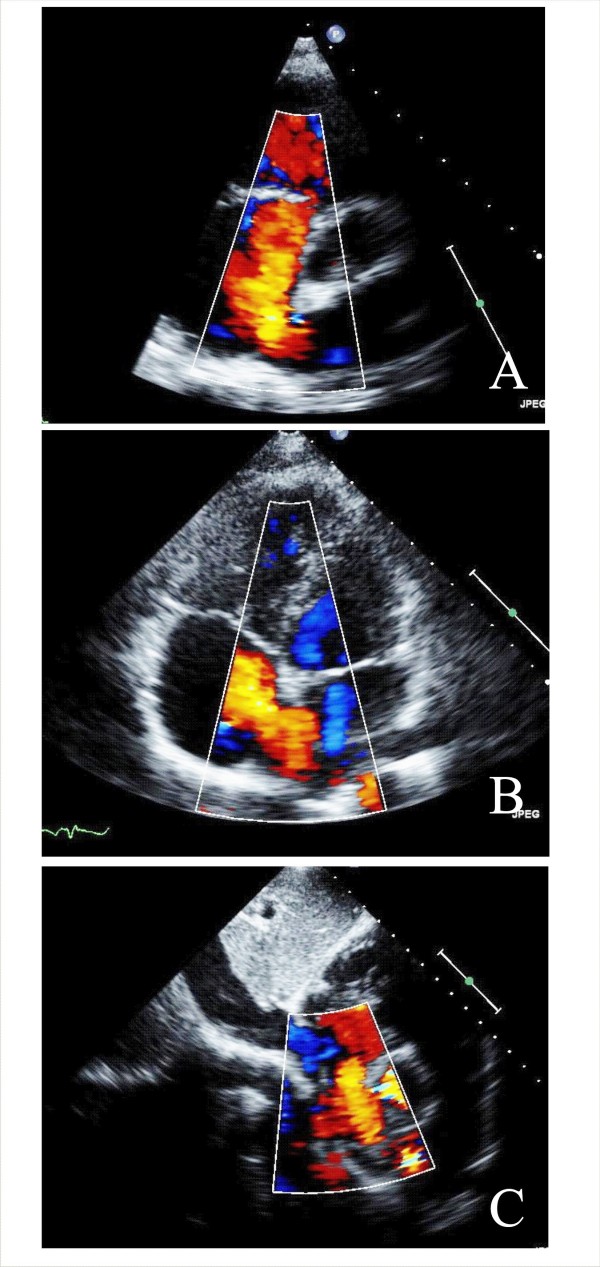
**Color flow Doppler shows left to right interatrial shunting at basal parasternal short axis (Panel A), at apical 4- chamber view (Panel B), and at subcostal 4-chamber view (Panel C)**.

The TTE protocol to imaging ASD is the same applied during the study of PFO (Figure 9) [3,51].

**Figure 10:**
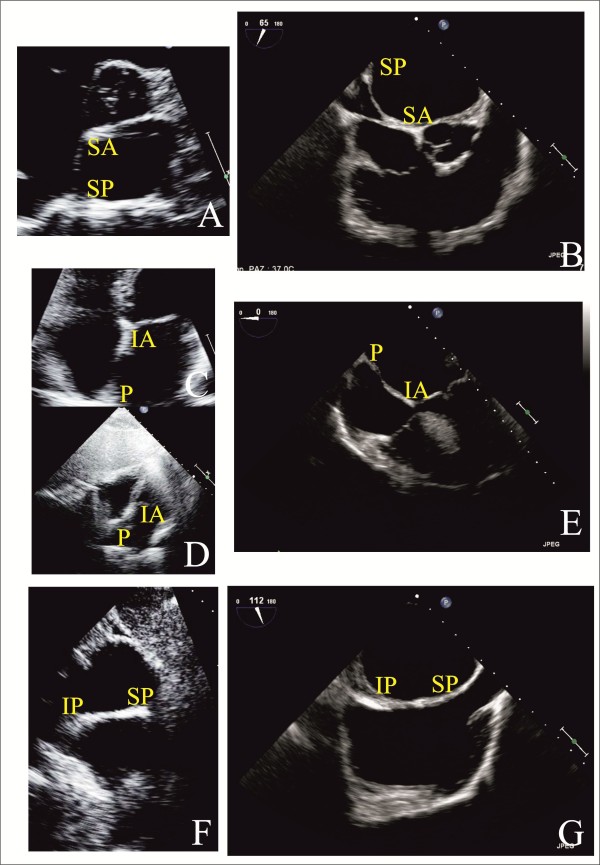
**ASD Rims by TTE and TEE: I**. TTE parasternal short axis at level of aortic valve view (Panel A); TEE basal short axis view (Panel B); TTE apical (Panel C) and subcostal four chamber (Panel D) views; TEE trasversal four chamber view (Panel E); TTE subcostal short-axis view (Panel F) and TEE long- axis for bicaval veins (Panel G). IA = Inferoanterior; IP = Inferoposterior; P = Posterior; SA = Superoanterior; SP = Superoposterior.

TTE can evaluate ASD rims although, especially in adults, TEE is mandatory (Figure 10) [51]. Right ventricular volume overload indicates that ASD has some hemodynamic consequences: in the extreme and rare case, Eisenmenger's physiology may result [3]. Quantification of shunt flow can be accomplished with calculation of ratio of pulmonary blood flow (Qp) to systemic blood flow (Qs) [51], that correlates significantly with cardiopulmonary functional improvement after transcatheter ASD closure [52].

### Indications to percutaneous ASD closure

ASD closure is indicated in presence of hemodynamic significance (Qp/Qs ≥ 1,5 and/or right chambers volume overload) or after a paradoxical embolic event (Table 4) [4]. Large ASDs (more than 40 mm in diameter), and/or ASDs with other associated congenital defects, and/or with inadequate septal rim (< 5 mm) are referred to surgery [4,5,53]. Clinical and echocardiographic indications and contraindications to percutaneous ASD closure are reported in Table 4.

**Table 4:** Indications to ASD Closure

CLINICAL INDICATIONS	CLINICAL CONTRAINDICATIONS	ECHOCARDIOGRAPHIC INDICATIONS	ECHOCARDIOGRAPHIC CONTRAINDICATIONS
Cryptogenic Strokes Symptoms	Severe Pulmonary Hypertension Recent Gastrointestinal Bleeding Contraindications to Antiplatelet or Anticoagulant Therapy Infection at the Time of Implantation	Qp/Qs ≥ 1,5 Right Atrial and/or Right Ventricular Enlargement	Other Congenital Heart Defects ASD Diameter > 40 mm Inadequate Septal Rims Close Proximity to Coronary Sinus or Inferior Vena Cava

### Echocardiography guidance of percutaneous PFO and ASD closure

**Figure 11:**
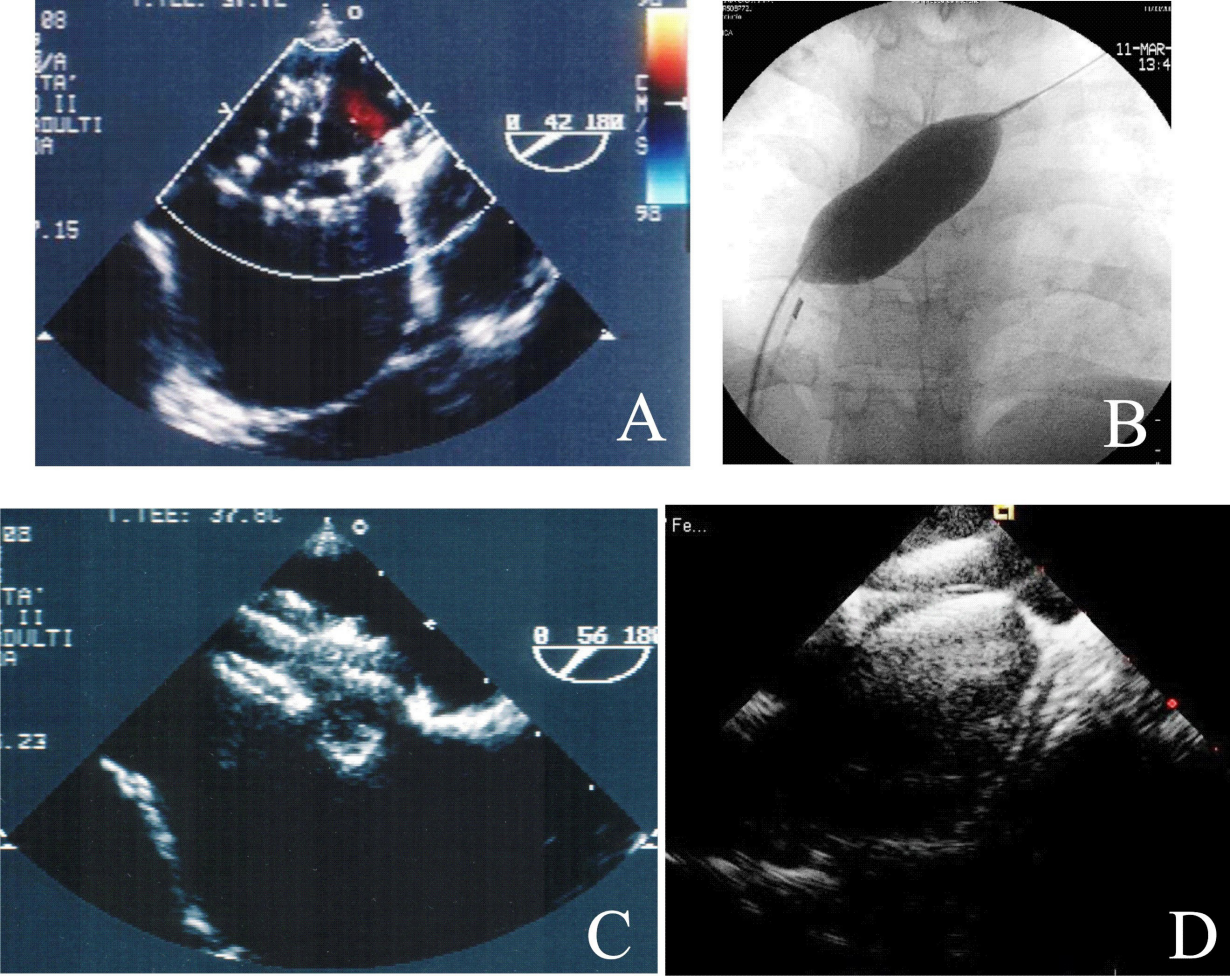
**Sizing of ASD by TEE (Panel A) and by fluoroscopy (Panel B); positioning of the device by TEE basal short axis view (Panel C) and by ICE long axis view (Panel D)**.

During the procedure for PFO, echocardiography is not usually required, although TEE guidance has been described extensively in adult patients [54]. ICE has been proposed as an alternative because does not need either conscious sedation or general anesthesia [54]. For PFO and ASD closure, there are a variety of atrial septal occluder devices. Mostly, devices for PFO closure "cover" the atrial septum, whereas devices for ASD closure "stent" it. During procedure, the defect is crossed with a curved catheter and semistiff wire; then, a catheter with balloon is inflated across the atrial septum until the complete occlusion of the ASD and absence of shunt is visualized (Figure 11). The distance between the two notches, viewed by fluoroscopy, is the diameter "stretched" of the defect, corresponding to the diameter of the device (Figure 11, Panel B). An appropriate diameter device is then positioned and deployed. Positioning and stability of the device, elimination of the shunt are evaluated by TEE or ICE (Figure 11) and fluoroscopy, and, if not satisfactory, the device can be retrieved at any time [5,9,34].

### Echocardiographic evaluation of PFO and ASD closure complications and follow-up

Percutaneous PFO and ASD closure is a safe and effective treatment in adults patients, even in case of thrombophilia or pulmonary hypertension, also during a long-term follow-up, up to 11 years [6,7]. Early post-procedure complications, such as pericardial tamponade has been reported in approximately 0.5% to 1% of patients; tamponade most frequently results from LAA perforation during the trans-septal guide wire anchoring [55].

A early or late post-procedure relatively rare complication is device embolization [56]. Residual shunts immediately after device closure of PFO/ASD, are common and often disappear or decrease, as the device endothelializes [55]. However, if shunts persist, serial TTE evaluation must be performed to follow-up the degree of shunting [55].

Erosion has been described, as late complication, in patients with multiple devices, deficient antero-superior rims and oversizing devices that, also, can cause superior vena cava occlusion [53,55].

Device thrombosis appears to be more common with devices containing uncoated metal arms, within the first month after device implantation [56]. Finally, a late rare complication is severe mitral valve insufficiency, probably due to oversized mismatched device traction on the root of the mitral annulus and mitral annular insufficient rim [56].

During follow up, TTE is recommended at 6, 12 and 24 months [56].

## Percutaneous closure of LAA in patients with AF

### Echocardiography assessment of LA cavity and LAA in patients with AF

Patients with AF have an increased risk of thrombembolic stroke [57] and in non valvular AF, LAA is the major site of thrombus formation [58,59].

**Figure 12:**
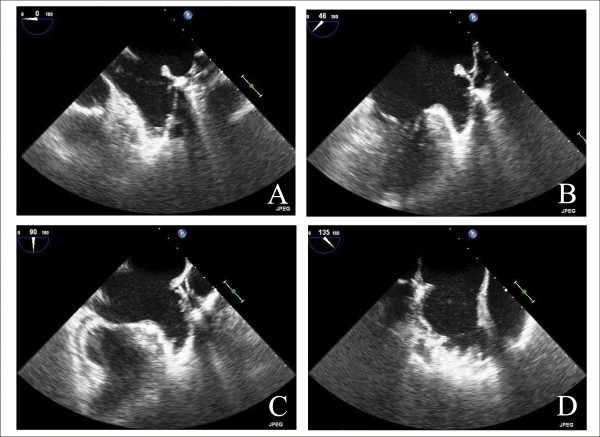
**TEE Image of LAA: horizontal short-axis view at the base of the heart, at 0° (Panel A), and 45° (Panel B), left two-chamber longitudinal view, at 90° (Panel C) and the view at 135° (Panel D)**.

LAA has a tubular form and is attached by a narrow junction to the left atrium [60], it is near to left superior pulmonary vein and it is closely associated with left aortic sinus. LAA orifice (ostium) is elliptical and located between the left ventricle and left superior pulmonary vein, extending over the atrioventricular groove and the surface of the left ventricle towards the left circumflex coronary artery [60]. TEE is the gold standard to exclude LA and LAA thrombus and to study LAA anatomic relationship. TEE views usually used to image LAA are reported in Figure 12. A multiplanar probe revolving around the cavity (0 to 180°) improves the assessment of its frequently complex structure [61].

### Indications to percutaneous LAA closure

Warfarin is the gold standard treatment to prevent embolic stroke, but benefits of anticoagulation do not come without risk of bleeding [62,63] and, moreover, it is contraindicated in up to 44% of patients with AF [63].

At present, percutaneous LAA closure is an acceptable option in selected patients with AF at high-risk of stroke who are not candidates to oral anticoagulation (Table 5). In addition, in patients on oral anticoagulation, LAA occlusion may reduce recurrence of stroke [64] (Table 5).

**Table 5:** Indications to LAA Percutaneous Closure

CLINICAL INDICATIONS	CLINICAL CONTRAINDICATIONS	ECHOCARDIOGRAPHIC INDICATIONS	ECHOCARDIOGRAPHIC CONTRAINDICATIONS
High Risk Patients with AF Not Candidates for Oral Anticoagulation Patients with Recurrent Strokes Despite on Oral Anticoagulation	Recent Surgical Procedure	LAA Depth ≥ 10 mm	Active Endocarditis or Bacteriemia Intracardiac Thrombus Close Association of LAA with Mitral Valve, Pulmonary Veins and Circumflex Artery

Clinical and echocardiographic indications and contraindications to percutaneous LAA closure are reported in Table 5.

### Echocardiography guidance of percutaneous LAA closure

Independently of the type of device, the actual deployment methodology is similar. First, standard transeptal puncture is performed for left atrial access. Next, the sheath is advanced up to the LAA orifice and a pigtail catheter is inserted into LAA. Then, TEE and fluoroscopic evaluate size (length and diameter), shape, and angulation of the orifice and body of the LAA. The device is then sized and is advanced into the LAA orifice [65].

Currently, three devices for LAA occlusion have been specifically designed: the Percutaneous LAA Transcatheter Occlusion (PLAATO), the WATCHMAN LAA system and Amplantzer Cardiac Plug Device. The PLAATO device was the first device, consisting of a self-expandible nitinol cage covered with a nonthrombogenic occlusive expanded polytetrafluoroethylene membrane and currently it is not available because of economic reasons. The WATCHMAN LAA System (Atritech, Plymouth, MN) consists of a parachute-shaped device with a self-expanding nitinol frame structure with a permeable polyester membrane over the atrial side and mid-perimeter fixation barbs to secure it in the LAA; it is permeable to blood, thus patients require conventional thromboembolic prophylaxis with 6 weeks of warfarin, at which time device endothelialization is confirmed by TEE [66]. More recently, the Amplatzer Cardiac Plug (ACP)(AGA Medical) is a self-expanding flexible braided nitinol mesh structure designed as a distal lobe and a proximal disc linked via a flexible central waist [66,67]; the lobe of the device is designed to conform to the inner wall of the LAA with a depth of 10 mm or more. For this device, TEE at 45°, measures the "landing zone" from the origin of left circumflex coronary artery to the roof of LAA, at least 10 mm below the ligament of Marshall (Figure 12, Panel B). The lobe of the device is anchored in the landing zone, 1-2 cm distal of the LAA orifice, while the disc fully covers the orifice of LAA. The size of the device should be at least 2 mm larger than the diameter of the LAA landing zone [67].

**Figure 13:**
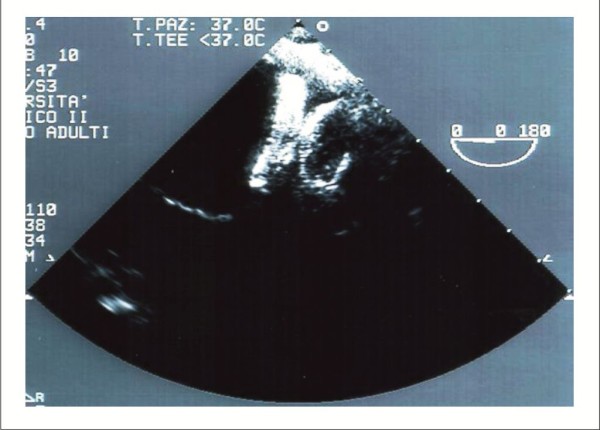
**EE Guidance of Percutaneous LAA Closure: evaluation of opening of the device**.

TEE is also used to determine any residual flow in the LAA and to examine device stability [67]. Adequacy of LAA occlusion can be assessed by contrast injection in the left atrium and the adequacy device stability by applying a tug that displaces device by 1 to 2 cm. When imaging confirms optimal positioning, device is deployed and delivery system is withdrawn into the right atrium (Figure 13, ICE is able to provide imaging support comparable to TEE, including LAA ostial size [68].

### Echocardiographic evaluation of LAA closure complications and follow-up

To date, LAA percutaneous closure, in particularly with Amplatzer device, have proved to be a procedure with a high success rate (96%) and low rate of serious complications (7%) [69].

**Figure 14:**
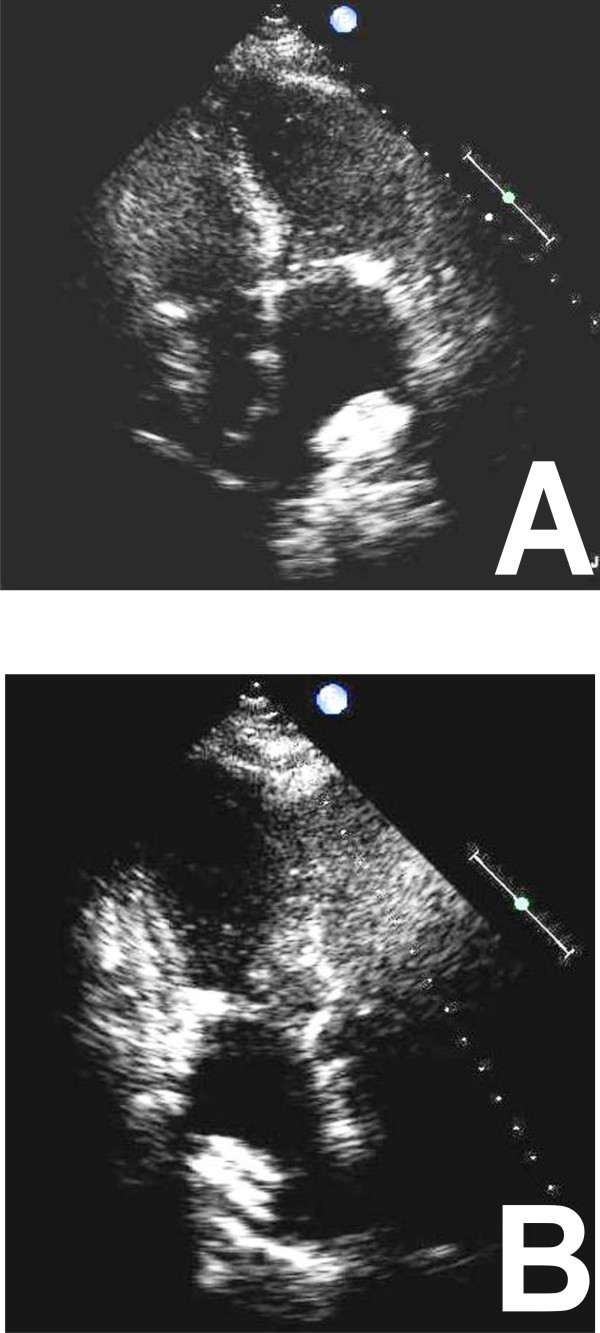
**TTE view of a LAA Amplantzer device migration in LA, by apical 4-chambers (Panel A) and 3-chambers (Panel B)**.

Complications include: ischemic stroke; pericardial effusions; tamponade; LAA tear/rupture and device embolization [65,67,69] (Figure 14). To verify the absence of these complications, a TTE follow up should be recommended at a day after procedure, at 4 weeks, 3 months and then every 6 months.

## Percutaneous closure of PVLs

### Diagnosis of PVLs

PVLs indicates a regurgitation between the prosthetic ring and the native valvular annulus [70-72] predisposed by endocarditis, annular calcification and redo operation for prosthesis malfunction [70].

**Figure 15:**
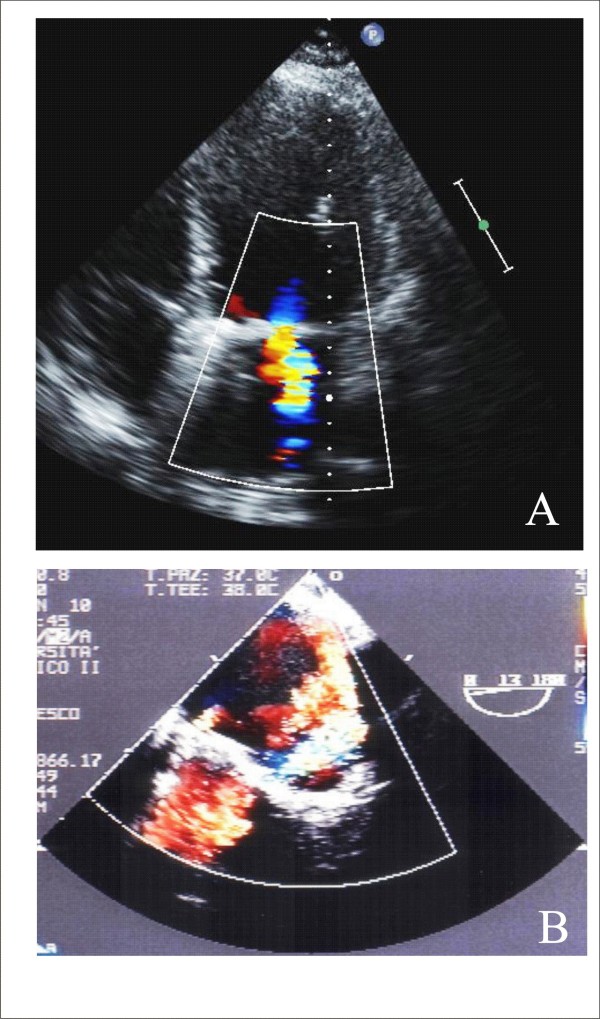
**Mitral PVL visualized by TTE apical view (Panel A) and by TEE trasversal four chamber view (0°) (Panel B)**.

TTE is the first diagnostic approach to PVLs (Figure 15) [70,73]. However, because mechanical mitral valve leaks produces LA shadowing, the degree of regurgitation is established by indirect Doppler signs of increased flow throughout the prosthesis [74]. In prosthesis aortic valve leaks, because the aortic does not create shadowing in the LVOT, TTE can estimate both the degree of regurgitation and the circumferential extent of the regurgitation > 20% [73]. To select patients for percutaneous treatment TEE is mandatory: TEE confirms presence, numbers, anatomy and position of mitral and aortic PVLs. Estimation of size and shape of the defect [75] is important in device selection, taking into account the irregular 3-dimensional structure of these tunnels [75]. TEE evaluation of mitral prosthetic valves is done by centering the prosthesis in the mid-esophageal four-chamber view. Then the sewing ring is imaged in full by rotation of the imaging plane from 0° to 180°, keeping the sewing ring in the center of the image and making small adjustments of the transducer tip. Anatomic landmarks for localization of paravalvular leakage usually used are the aorta and LAA [71]. For aortic prosthesis, TEE complements TTE mostly in posterior PVLs. TEE may be limited in evaluating prosthetic AR in the midesophageal level because of anterior shadowing. In case of mitral and aortic prosthesis it is critical to evaluate the aortic one from transgastric view [71].

### Indications to percutaneous PVLs closure

Patients with severe PVLs and/or haemodynamic instability with symptoms of congestive heart failure or severe hemolysis are candidates to leak correction [71,76]. Surgery is the gold standard therapy, though reoperation is associated with a markedly higher morbidity and mortality, and higher risk of paravalvular leakage than the initial procedure [76,77]. In patients not candidates for surgery, percutaneous closure has become a feasible option (Table 6) [76]. Clinical and echocardiographic indications and contraindications to percutaneous closure are reported in Table 6.

**Table 6:** Indications to PVLs Closure

CLINICAL INDICATIONS	CLINICAL CONTRAINDICATIONS	ECHOCARDIOGRAPHIC INDICATIONS	ECHOCARDIOGRAPHIC CONTRAINDICATIONS
Symptoms of Congestive Heart Failure in Patients Not Candidates for Surgery Severe Hemolysis in Patients Not Candidates for Surgery	Severe Pulmonary Hypertension	Adequate Echocardiographic Visualization	Active Infection or Vegetations Unstable Rocking Valve Intracardiac Thrombus Large Leaks Leaks Close to the Point of Maximum Leaflets Excursion

### Echocardiographic guidance of PVLs percutaneous closure

**Figure 16:**
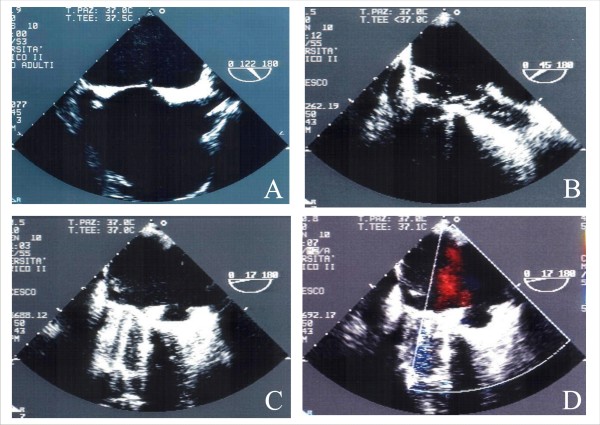
**TEE evaluation of Mitral PVL Percutaneous Closure: Transeptal puncture (Panel A); mitral prosthesis is crossed with an atraumatic guide-wire (Panel B); positioning of the device (Panel C) and absence of a residual leak (Panel D)**.

TEE is commonly used for image guidance [73]; ICE may represent an alternative, though experience with TEE is greater [76]. Mitral PVLs can be approached retrogradely from a catheter in the left ventricle [76]. In aortic PVLs, the same approach is recommended, however, it must be carried out very quickly because may induce prosthetic dysfunction [78]. In the mitral anterograde approach, a transeptal puncture is performed in the standard fashion (Figure 16, Panel A) [72]. The defect of mitral or aortic prostheses is crossed with an atraumatic guide-wire (Figure 16, Panel B). Sonographer should attempt to identify a cardiac structure in proximity to the leak utilized as an initial point of radiographic reference for the interventionist. Once a catheter is in the central region of the leak, small injections of saline, containing sufficient micro-bubbles, are used to assist in echocardiographic localization of the catheter tip and to guide manipulation [76]. Wire passage through the leak can usually be visualized by echocardiography, and entrapment in ventricular trabeculations, mitral, or tricuspidal chordate may be appreciated [76]. The choice of available device is dependent on the specific size and morphology of the PVLs as well as on the proximity to prosthetic leaflets. No specific designed devices but umbrella devices, vascular occluding devices, and coils, have been implanted [72]. Finally, a closure device is introduced and the device deployment and positioning may be assessed in real time (Figure 16, Panel C and D); only when echo confirms correct positioning, stability, and absence of interference with normal valve function, the occlusive device is released [72,76].

### Echocardiographic evaluation of PVLs closure complications and follow-up

PVLs percutaneous closure is a feasible procedure in selected patients, with a reasonable degree of technical and clinical success, complete or partial PVL occlusion in 70% of patients, with concomitant clinical improvements and an acceptable level of serious complications (10%) [76].

Early complications associated with device closure of PVLs include incomplete closure, impairment of valvular function, embolization and onset of new haemolysis [9].

A major limitation of perivalvular leak closure is the frequent persistence of leak, often due to the semilunar shape of the defects. In such cases, it may be prudent to plan a staged procedure, first implanting one device and after this has fibrosed, deploying another, rather than risking dislodgement or embolization of the first device with immediate implantation of a second [76,79]. After the procedure, hemolysis may develop due to flow through the device, resolving with time as the device thromboses [76], or because a successful reduction in PVLs size, increases shear forces across the narrower orifice.

To ensure that device has not migrated, the leak has been closed follow-up with TTE is generally adequate at 1, 6 and 12 months.

## Conclusions

Significant advances in percutaneous repair of many heart diseases have highlighted the importance of a systematic approach for selection, guidance and follow up of patients undergoing these procedures.

Echocardiography plays a critical role in patient selection, particularly in choosing the appropriate size of the prosthesis to be implanted. To date, TAVI is targeted at high-risk patients but it may be extended to the lower risk group in the future, if the initial promise holds true after careful evaluation.

A combination of TEE and supplemental TTE has been used for MitraClip procedure. Its safety profile is similar to other percutaneous procedures and now it is attractive for high-risk surgical candidates, in future randomized trial results will define the role for surgical candidates.

Understanding correlation between anatomy and echocardiography is perhaps the most essential requisite to ensure a successful PFO/ASD percutaneous closure procedure. The complication rates for both TEE and ICE imaging to guide this procedures appear to be low and acceptable, but ICE should be considered when suitable expertise is available. LAA percutaneous closure is an emerging approach and it, with appropriate patient selection, device iterations, and technical improvements, may become an important viable therapeutic alternative to chronic antithrombotic therapy. PVLs percutaneous closure is a new approach that can be useful for increasing PVLs because of increasing valve replacements. Appropriate designed devices for PVLs closure and improved imaging, particularly with ICE and with a wide use of 3D TEE, will help in this procedure. The echocardiography has an important and undeniable role. For the best result of percutaneous treatment, a close collaboration between sonographers and interventional cardiologists is required.

## Consent

Written informed consent was obtained from the patient for publication of this report and any accompanying images.

## Abbreviations

AF: Atrial Fibrillation; AHA/ACC: American Heart Association/American College of Cardiology; AHA/ASA: American Heart Association/American Stroke Association; AR: Aortic Regurgitation; AS: Aortic Stenosis; ASA: Atrial Septal Aneurysm; ASD: Atrial Septal Defects; CDS: Clip Delivery System; CT: Computed Tomography; ESC: European Society of Cardiology; ESO: European Stroke Organisation; EuroSCORE: European System for Cardiac Operative Risk Evaluation; EVEREST: Endovascular Valve Edge-to-Edge Repair Study; ICE: Intracardiac Echocardiography; LA: Left Atrium; LAA: Left Atrial Appendage; LV: Left Ventricle; LVOT: Left Ventricular Outflow Tract; MR: Mitral Regurgitation; PFO: Patent Foramen Ovale; PVLs: Para-Valvular Leaks; Qp: Pulmonary Blood Flow; Qs: Systemic Blood Flow; STS score: Society of Thoracic Surgeons Cardiac Operative Risk Evaluation; TAVI: Transcatheter Aortic Valve Implantation; TCD: Transcranial Doppler; TEE: Transesophageal Echocardiography; TTE: Transthoracic Echocardiography; Valve in Valve procedure: percutaneous implantation of a second device inside the primary percutaneous aortic prosthesis.

## Competing interests

The authors declare that they have no competing interests.

## Authors' contributions

C Contaldi drafted the manuscript; MA Losi designed the paper; A Rapacciuolo, G Esposito, F Piscione, MA Losi and S Betocchi revised it critically; R Lombardi, V Parisi, M Prastaro, LS Parrella, A Giamundo, C Di Nardo and R Puglia analyzed data from literature. All authors have read and approved the manuscript.
